# Kinematic analysis of the daily activity of drinking from a glass in a population with cervical spinal cord injury

**DOI:** 10.1186/1743-0003-7-41

**Published:** 2010-08-20

**Authors:** Ana de los Reyes-Guzmán, Angel Gil-Agudo, Benito Peñasco-Martín, Marta Solís-Mozos, Antonio del Ama-Espinosa, Enrique Pérez-Rizo

**Affiliations:** 1 Biomechanics and Technical Aids Unit, National Hospital for Spinal Cord Injury. SESCAM, Finca la Peraleda s/n, Toledo, Spain

## Abstract

**Background:**

Three-dimensional kinematic analysis equipment is a valuable instrument for studying the execution of movement during functional activities of the upper limbs. The aim of this study was to analyze the kinematic differences in the execution of a daily activity such as drinking from a glass between two groups of patients with tetraplegia and a control group.

**Methods:**

A total of 24 people were separated into three groups for analysis: 8 subjects with metameric level C6 tetraplegia, 8 subjects with metameric level C7 tetraplegia and 8 control subjects (CG). A set of active markers that emit infrared light were positioned on the upper limb. Two scanning units were used to record the sessions. The activity of drinking from a glass was broken down into a series of clearly identifiable phases to facilitate analysis. Movement times, velocities, and the joint angles of the shoulder, elbow and wrist in the three spatial planes were the variables analyzed.

**Results:**

The most relevant differences between the three groups were in the wrist. Wrist palmar flexion during the back transport phase was greater in the patients with C6 and C7 tetraplegia than in the CG, whereas the highest wrist dorsal flexion values were in forward transport in the subjects with C6 or C7 tetraplegia, who required complete activation of the tenodesis effect to complete grasping.

**Conclusions:**

A detailed description was made of the three-dimensional kinematic analysis of the task of drinking from a glass in healthy subjects and in two groups of patients with tetraplegia. This was a useful application of kinematic analysis of upper limb movement in a clinical setting. Better knowledge of the execution of this movement in each of these groups allows therapeutic recommendations to be specifically adapted to the functional deficit present. This information can be useful in designing wearable robots to compensate the performance of AVD, such as drinking, in people with cervical SCI.

## Background

Upper limb functionality is fundamental for the execution of basic activities of daily living (ADL) like drinking, eating and personal hygiene. Impaired upper limb function is one of the most common sequelae in central nervous system injury [1,2]. In the case of spinal cord injury (SCI), the upper limb is affected in more than 50% of cases [3]. Upper limb strength is impaired to some extent in people who have suffered cervical SCI, making it difficult for them to perform many ADLs essential for their autonomy. They may require technical assistance. Therefore, these patients experience sharp limitations in their level of activity and participation in the social setting, as people who have suffered another central nervous system injury, such as stroke [4].

Until now, upper limb function has been evaluated using a series of scales, such as the Fugl-Meyer Assessment, Frenchay Arm Test, Motor Assessment Scale, Box and Block Test, and the Nine-Hole Peg Test [5,6]. These tools are sensitive to gross functional changes, but less sensitive in measuring small and more specific changes [7]. Moreover, the use of these scales is not exempt from a degree of subjectivity.

In contrast with the lower limb, the upper limb has extensive functionality due to the mobility of numerous joints that can execute fine movements thanks to complex neuromuscular control [7]. For that reason, objective measurement elements and exact systems of movement analysis are necessary to be able to describe upper limb activities more precisely and specifically. Biomechanical analysis and, specifically, kinematic analysis techniques are interesting tools for obtaining objective data. At present, complex systems of kinematic analysis allow the automated analysis of movement in three dimensions. The biomechanical model of the lower limb has been implemented for most equipment because gait is one of the movements most analyzed by biomechanics laboratories. Consequently, in order to analyze the upper limb it is necessary to previously define and develop the biomechanical model based on the activity to be analyzed.

Kinematic studies have been made of the upper limb in which reaching/grasping movements on a horizontal plane as a free movement without arm support [8] and with arm support [9-11] have been analyzed. However, the analysis of purpose-oriented movements must be proposed because the musculoskeletal system has potentially a larger number of ways to achieve the motor task, permitting the organism to adapt to different environmental conditions. So, the musculoskeletal system takes advantage of this feature of the motor apparatus by selecting a desired trajectory and an interjoint coordination among many possible strategies to make goal-oriented movements [12-14]. Studies have been published on kinetic analysis of the shoulder and elbow in healthy subjects performing a set of ADLs [15,16] and on complete kinematic analysis of the upper limb during the movement of drinking from a glass [7].

It has been confirmed that the characteristics of movement can vary depending on the objective to be completed. For example, the kinematics of the upper limb is not the same in pointing to an object as when a grasping function is added [11,17,18].

Several studies have been published recently on the three-dimensional analysis of ADLs in healthy subjects [7,8,19,20]. Similar studies have been made in patients with different neurological conditions [21-23]. Although there have been few reports in patients with SCI, the results of the kinematics of grasping and the movements of pointing toward an object in patients with C6 tetraplegia have been described [24]. However, we found no studies of the kinematic analysis of the upper limb when performing a functional activity like drinking from a glass in patients with different levels of cervical SCI.

One working hypothesis has been that differences are likely to exist between people with cervical SCI and people without such an injury. On the other hand, the different levels of injury affect the upper limb musculature differently and such differences should be manifested by their respective movement patterns when executing the drinking task. Identification of the different mobility patterns could be useful in clinical practice to set therapeutic goals appropriate to the severity of the injury.

Consequently, the objectives of the present study were:

1. To compare the data obtained from kinematic analysis of the upper limb during the drinking task in people with cervical SCI and a control group.

2. To compare the data obtained by kinematic analysis of the upper limb during the drinking task between people with two different levels of cervical SCI.

## Methods

### Population

Twenty-four subjects divided into three groups were included in this study: a control group (CG), subjects with metameric level C6 tetraplegia (C6 group) and subjects with metameric level C7 tetraplegia (C7 group). Each group contained 8 subjects. The demographic and anthropometric characteristics of the CG were similar to those of the two groups of patients with SCI (Table 1). All subjects were right-handed. In the case of subjects with C6 and C7 tetraplegia, the etiology of injury was trauma in every case. The patients screened had to fulfill the following criteria to be included in the study: age 16 to 65 years, injury of at least 6 months' duration and level of injury C6 or C7 classified according to the American Spinal Injury Association (ASIA) [25] scale into grades A or B. Patients who presented any vertebral deformity, joint restriction, surgery on any of the upper limbs, balance disorders, dysmetria due to associated neurologic disorders, visual acuity defects, cognitive deficit, or head injury associated with the SCI were excluded. The subjects were classified into C6 or C7 tetraplegia by a physical examination. The upper limb Motor Index was obtained [25], with the assessment of the strength of five muscle groups of the right upper limb by a physiotherapist. Each muscle group can be evaluated between 0 (no function)-5 (normal function) with a total of 25 points. All patients signed an informed consent form before the study. The guidelines of the declaration of Helsinki were followed in every case and the study design was approved by the local ethics committee.

**Table 1:** Demographic and functional characteristics of the sample analyzed (n = 24)

	Control group (n = 8)	C6 group (n = 8)	C7 group (n = 8)
**Sex (male)^†^**	3	4	5
Age (years)*	29.50 (4.00)	33.63 (13.03)	28.75 (9.82)
Height (cm)*	167.4 (3.24)	172.5 (8.91)	176.2 (8.89)
Weight (kg)*	62.00 (7.07)	70.25 (7.10)	68.37 (12.18)
Length of the right arm (cm)*	57.07 (2.34)	58.03 (3.21)	58.37 (3.90)
**Injury etiology (Traumatic)^†^**	-	8	8
Months since injury*	-	8.50 (2.20)	7.50 (1.85)
**ASIA (A)^†^**	-	3	3
**ASIA (B)^†^**	-	5	5
Index Motor right arm (0-25)*	25.00 (0)	12.00 (2.07)	14.12 (2.03)

* Mean and standar desviation for continuous variables

† n for categorical variables

### Movement recording system and markers

Three-dimensional movement capture was recorded with CodaMotion equipment (Charnwood Dynamics, Ltd, UK). This equipment has active markers that emit infrared light, which was recorded by two scanning units in this study. The marker images are displayed on a computer screen and projected as X, Y, and Z coordinate values.

**Figure 1:**
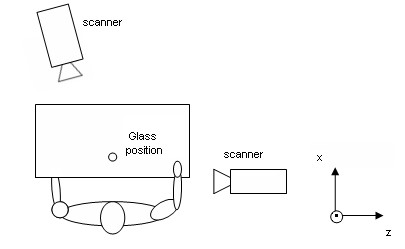
**View from above of the set-up for the activity of drinking from a glass**. The XYZ coordinate system is visible. The subject has the arm at the starting point.

One of the cameras was placed in front of the table, slightly to one side with respect to midline and contralateral to the study side of the subject. The other camera positioned laterally (Figure 1). The system was calibrated by placing three active markers on the floor to serve as the laboratory reference system. The coordinate system was defined with the X-axis directed forward (anteriorly), the Y-axis upward (superiorly) and the Z-axis to the side (laterally) [26]. The location of the cameras and markers was validated with a person sitting in the measurement area to ensure that the markers were recorded by least by one of the cameras throughout the drinking activity.

**Figure 2:**
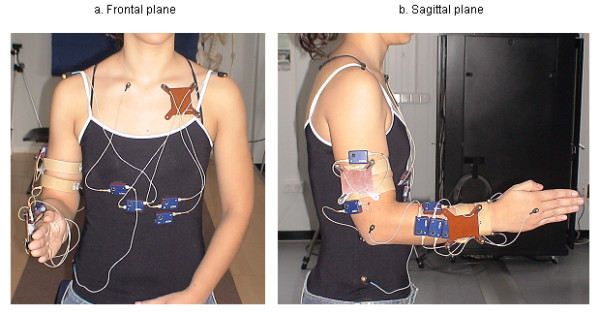
**Actual marker positions on the subject**. Figure show a) a frontal plane view (Y-Z) and b) a sagittal plane view (X-Y).

Eighteen markers were used. Following the recommendations of earlier studies, the body segments were defined by placing 8 markers on the superficial bony prominences of the right upper limb, which were easily positioned in the different analyses [7,10,12,27,28]. These markers were placed on the head of the third metacarpal, radial and ulnar styloid processes of the wrist, lateral and medial epicondyles of the elbow, right and left acromion and right iliac crest. The biomechanical model of upper limb movement was completed with another 10 markers mounted on rigid pieces that were placed on each body segment. These pieces were used with the aim of minimizing any error originated by possible marker displacement on the skin. These pieces had to be light, comfortable for the subject to wear, and had to be fixed onto points where the least amount of movement was possible [22]. Four markers were placed on the chest, three mounted on a support and one directly on the skin; three markers mounted on a support placed on the arm, and the last three markers mounted on a support placed on the forearm (Figure 2). The final position of the last 10 markers and the position of the cameras was the position that yielded the best marker visibility to the scanning cameras during the movement of drinking from a glass and the best measurement results in the processed recordings.

### Experimental set-up and procedure

All subjects were right-handed and performed the movement of the drinking task with the right arm. Subjects with C6 or C7 tetraplegia sat in their own wheelchairs and the control subjects sat in a conventional wheelchair, Action3 Invacare (Invacare Corp, Elyria OH, USA) with a configuration similar to that of the wheelchair of the subjects with tetraplegia. The chair was placed before a table measuring 120 × 60 × 72 cm. In every case, the subject-to-table distance was 18-20 cm and the angle between the seat and back was 90-100°. The starting position (position of calibration) for all the subjects was defined as a position in which the subject's trunk rested firmly against the back of the chair. All subjects put their feet on the footrests with a foot-leg angle of 90°. The right upper arm was placed against the trunk and the elbow was flexed 90° flexion and in a neutral pronation-supination, i.e., with the palm of the hand perpendicular to the table surface and facing inward (medially). The ulnar side of the wrist rested close to the surface of the table (Figure 1). In every case, the sitting and table heights could be adapted with the aim of obtaining the same starting position for all the subjects. The subject rested the left hand on the lap. A hard plastic glass measuring 6.5 cm in diameter by 17.5 cm high was used. It was filled with 1 dl of water and placed 18 cm from the edge of the table where the subject was seated, in the area marked on the table (Figure 1).

Each subject received an explanation about how to perform the drinking task, which consisted of reaching out for the glass from the starting position and grasping it, raising the glass to the mouth, drinking, lowering the glass to the pickup point, and returning the hand to the starting position. All the subjects practiced the activity twice to find a comfortable sitting position before the movement exercise was recorded. This test confirmed that the subjects could carry out the activity. Once this phase was completed satisfactorily, a static calibration recording was made. Using the static calibration recording, we checked that each marker was visible to at least one of the scanning cameras at all times. Movement recordings were made as the subject executed the drinking task at a comfortable, self-selected speed. Before a recording was accepted as validated, we checked it to ensure that the markers were visible at all times. Five valid recordings of each subject were obtained for analysis and processing.

The consistency and repeatability of the test protocol was assessed by conducting a test-retest sequence with four randomly selected control subjects. Test-retest involved recording the action of the drinking task and then removing the markers. The entire procedure was repeated from the beginning fifteen minutes later.

### Data processing

The recordings were processed with Visual 3D software (C-Motion, Inc., USA), which involved using a signal processing program to obtain signals of the movement of different joints at a sampling frequency of 200 Hz, the maximum allowed for the 18 markers used with the two scanning units. Signals were filtered using a low-pass Butterworth filter with a cutoff frequency of 1.5 Hz. The three best recordings were selected from the five recordings made on the basis of best marker visibility in each recording. The mean of these three recordings yielded the final measurement value for each subject. The human arm was modeled for three-dimensional kinematic analysis in three segments, the arm, forearm and hand, which were considered as rigid solids [29]. A local coordinate system was defined for each segment following the recommendations of the International Society of Biomechanics [26]. In the arm, the origin of the reference system was at the center of the glenohumeral joint, 2 cm below the acromion. Also, the Y-axis corresponded to the line that joined the midpoint between the lateral and medial epicondyles and the center of the glenohumeral joint in proximal direction and the Z-axis was the mediolateral axis pointing to the right. In the forearm, the origin was at the midpoint between both epicondyles of the elbow, the Y-axis was formed by the line that joined the midpoint between the radial and ulnar styloid processes with the midpoint between the lateral and medial epicondyles proximally and the Z-axis was the line that joined both epicondyles in the lateral direction. In the hand, the origin was at the midpoint between radial and ulnar styloid of the wrist, the Y-axis was the line joining the head of the third metacarpal with the midpoint between the radial and ulnar styloid processes proximally and the Z-axis joined both styloid processes laterally. We obtained trunk movement with respect to the laboratory coordinate system, arm movement with respect to the trunk, forearm movement with respect to the arm, and hand movement with respect to the forearm using Euler angle notation and a sequence of ZXY rotations of the trunk, arm and hand, and ZYX rotations of the forearm.

In each recording, a complete cycle of the drinking task was identified. The beginning of the cycle was the onset of displacement of the marker on the head of the third metacarpal and the end of the cycle was the return of the marker to the starting point after completing the drinking task. As it happens with other cyclical movements, such as walking, several phases were established in the drinking task to facilitate task analysis. We used phases and events delimiting the phases that have been described previously: reaching, forward transport, drinking, back transport and return [7].

Once the recordings were made and analyzed, the results were described in terms of analysis of the following variables:

• Movement times: the duration of each phase and the complete cycle.

• Peak velocities: the velocities were obtained by calculating the linear velocity with which the hand moves in the phases of the cycle of reaching, forward transport, back transport and return to start position.

• Joint angles: flexion-extension and lateral inclination of the trunk; flexion-extension, abduction-adduction and external-internal rotation of the shoulder joint; flexion-extension and pronation-supination of the elbow joint; and dorsal-palmar flexion of the wrist. For each joint angle, we calculated the maximum, minimum, range of motion (ROM) and moment in the complete drinking cycle in which these values were reached.

• Coordination between the shoulder and elbow joints, particularly between the shoulder flexion angle and the elbow flexion angle, in the reaching phase.

In order to compare the three groups analyzed, the duration of the cycles was adjusted for time and expressed as percentages. Consequently, data were expressed in relation to the percentage of the drinking task cycle that had lapsed (0-100% of the drinking task cycle) when the movement was recorded.

### Statistical analysis

A descriptive analysis was made of the clinical and functional variables by calculating the median and interquartile range of the quantitative variables and the frequencies and percentages of the qualitative variables.

Given the limited number of participants, non-parametric methods were used. The Kruskal-Wallis test was used to find possible differences in each variable between the three groups analyzed; the Kruskal-Wallis test began by testing the equivalence hypothesis between groups. If the significance of the Kruskal-Wallis test is p < 0.05, the equivalence of behavior between groups can be rejected and a pairwise comparison can be made using the Mann-Whitney test. The Bonferroni correction was applied, which takes into account randomness due to multiple comparisons.

The interrelation between the shoulder flexion angle and the elbow flexion angle was analyzed in the reaching phase using the Pearson correlation coefficient.

The repeatability of the protocol was evaluated with the Student t test using a level of significance of 0.05. The mean difference between the test and retest values was calculated for each of the four control subjects in which test-retest consistency was analyzed with a 95% confidence interval.

Data were analyzed statistically using the SPSS for Windows (version 12.0) statistical package (SPSS Inc, Chicago, IL, USA).

## Results

### Movement times

The duration of the complete cycle of the drinking activity was more prolonged in the group of subjects with C6 tetraplegia than in CG (p < 0.05). In the phase analysis the duration of the reaching phase was shorter in CG than in subjects with C6 (p < 0.01) and C7 tetraplegia (p < 0.05) (Table 2). In both groups of subjects with tetraplegia, not only was the reaching phase of longer duration than in CG, it was also the longest phase in the cycle. In the CG the longest phase in the cycle was the back transport phase. The contribution of each phase to the complete drinking task cycle in each analyzed group is detailed in Tables 2.

**Table 2:** Duration of each phase of the drinking task cycle and peak velocities

	Control group (n = 8)		C6 group (n = 8)		C7 group (n = 8)	
**Kinematic variables**	**Median (IR)**	**% mov time** **Median (IR)**	**Median (IR)**	**% mov time** **Median (IR)**	**Median (IR)**	**% mov time** **Median (IR)**
						
**Movement times (s)**						
Reaching (+ grasping)	**1.04 (0.33)^a, c^**	**16.69 (3.86)^b, d^**	**2.57 (0.98)^c^**	**26.73(12.42)^b^**	**1.66 (1.07)^a^**	**21.83 (8.46**)**^d^**
Forward transport	0.91 (0.36)	**31.55 (10.04)^a, b^**	1.20 (1.40)	**41.76(10.97)^a^**	1.28 (0.81)	**40.24 (8.46)^b^**
Drinking	1.37 (1.20)	54.97 (6.42)	1.96 (2.07)	59.75 (7.85)	1.41 (0.55)	54.48 (8.74)
Back transport	1.42 (0.39)	79.86 (4.92)	1.70 (0.96)	80.79 (5.58)	1.62 (1.18)	78.15 (8.24)
Returning	**1.28 (0.25)^a^**	100 (0)	**1.73 (0.68)^a^**	100 (0)	1.61 (0.62)	100 (0)
Total movement time	**5.87 (1.80)^a^**	100 (0)	**8.52 (5.16)^a^**	100 (0)	7.63 (4.25)	100 (0)
						
**Peak velocity (PV) (m/s)**						
PV for reaching	0.66 (0.09)	**5.68 (1.80)^a, c^**	0.56 (0.26)	**2.57 (1.26)^c^**	0.67 (0.53)	**3.42 (1.91)^a^**
PV for forward transport	**0.69 (0.23)^a^**	**22.07 (4.53)^c, d^**	**0.44 (0.24)^a, b^**	**32.69(10.75)^c^**	**0.58 (0.60)^b^**	**30.51 (13.11)^d^**
PV for back transport	**0.72 (0.14)^a^**	63.39 (3.98)	**0.54 (0.15)^a, c^**	67.60 (6.52)	**0.79 (0.43)^c^**	60.73 (9.62)
PV for returning	0.63 (0.08)	**87.82 (4.68)^c^**	0.52 (0.13)	**92.46 (3.78)^c^**	0.52 (0.20)	89.40 (7.78)
						
**Joints Coordination**						
Pearson index value	-0.95(0.04)		-0.91 (0.11)		-0.95(0.08)	

a, b (p < 0.05, with Bonferroni correction)

c, d (p < 0.01, with Bonferroni correction)

Joints Coordination: Coordination between shoulder and elbow flexion movements

Abbreviations: IR- Interquartile range

% mov time-% of total movement time

### Peak velocities

The peak velocities of the forward and back transport phases were slower in subjects with C6 tetraplegia than in subjects with C7 tetraplegia (p < 0.05 and p < 0.01, respectively) and in CG (p < 0.05) (Table 2). In addition, the peak velocity of the reaching phase was reached later in controls than in the subjects with C6 tetraplegia (p < 0.01) and C7 tetraplegia (p < 0.05). In contrast, the peak velocity of the forward transport phase was delayed in the subjects with C6 tetraplegia (p < 0.01) and C7 tetraplegia (p < 0.01) compared to CG (Table 2).

### Joint angles

**Figure 3:**
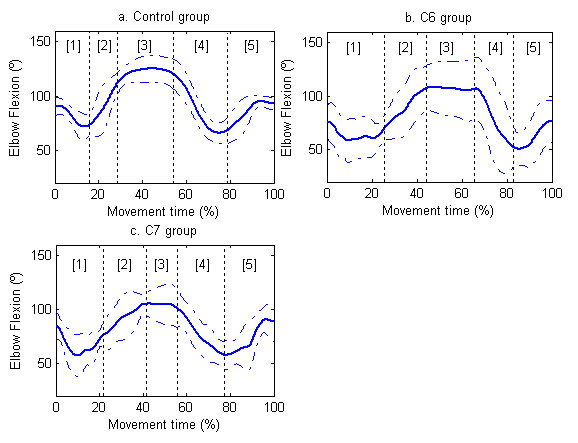
**Elbow flexo-extension**. Joint angles for elbow (extension-downward, flexion-upward). 0 degrees is considered as full extension. Figures 3a, 3b and 3c show the mean (continue thick line) and standard deviation (dashed line) of the CG, subjects with C6 tetraplegia and subjects with C7 tetraplegia, respectively. The vertical lines delimit the duration of the phases for each group [1] reaching, [2] forward transport, [3] drinking, [4] back transport, and [5] return to beginning.

In the shoulder joint, only the minimum abduction angle was greater in CG than in subjects with C7 tetraplegia (p < 0.01) (Table 3). In the elbow joint, the peak minimum of flexion angle was smaller in the subjects with C6 tetraplegia (p < 0.05) and C7 tetraplegia (p < 0.05) than in CG, but none of the differences in the elbow flexion-extension ROM of the three groups was statistically significant (Table 3 and Figure 3). None of the joint angles analyzed in the trunk showed significant differences (Table 3).

**Table 3:** Angles of joints analyzed during the cycle of drinking task

	Control group (n = 8)		C6 group (n = 8)		C7 group (n = 8)	
**Kinematic** **variables**	**Median (IR)**	**% mov time** **Median (IR)**	**Median (IR)**	**% mov time** **Median (IR)**	**Median (IR)**	**% mov time** **Median (IR)**
						
***Shoulder ***						
Max. Flexion	52.55 (21.31)	51.03 (13.60)	52.47 (11.29)	55.92 (10.92)	61.16 (22.77)	52.40 (8.66)
Min. Flexion	-4.48 (10.70)	97.89 (3.34)	0.21 (28.41)	99.86 (2.02)	-0.24 (11.01)	96.98 (8.45)
Range	59.53 (29.80)		45.49 (29.61)		63.75 (23.31)	
						
Max. Abduction	29.77 (10.67)	83.67 (49.55)	23.00 (18.71)	60.35 (43.95)	29.37 (27.17)	69.15 (32.33)
Min. Abduction	**13.55 (5.86)^c^**	13.35 (69.75)	8.40 (9.33)	28.08 (59.71)	**0.13 (10.84)^c^**	13.51 (21.67)
Range	16.61 (14.98)		15.80 (14.79)		25.01 (16.19)	
						
Max. Ext. Rot	-4.74 (4.77)	95.68 (5.92)	-9.90 (35.05)	91.13 (31.23)	-16.98 (9.54)	97.15 (7.32)
Max. Int. Rot	-45.59 (15.75)	68.62 (38.79)	-38.71 (27.81)	48.42 (32.78)	-48.89 (20.86)	63.81 (31.19)
Range	41.27 (8.29)		28.17 (17.06)		31.49 (36.22)	
						
***Elbow***						
Max. Flexion	128.20 (21.10)	**46.25 (7.93)^a^**	113.40 (33.65)	**53.84 (14.83)^a^**	114.05 (16.45)	45.30 (15.86)
Min. Flexion	**64.42 (11.75)^a, b^**	77.27 (39.16)	**42.54 (27.51)^a^**	79.15 (58.05)	**42.11 (20.94)^b^**	75.58 (27.94)
Range	64.86 (27.45)		70.85 (20.64)		67.30 (26.56)	
						
Max. Pronation	40.25 (22.37)	50.06 (4.79)	58.03 (33.33)	60.73 (30.52)	45.90 (23.73)	52.97 (16.02)
Min. Pronation	9.53 (22.75)	35.96 (48.62)	8.84 (22.20)	37.10 (58.00)	-3.62 (22.60)	37.13 (49.71)
Range	33.07 (14.64)		55.17 (29.61)		49.59 (12.66)	
						
***Wrist***						
Max. Palmar Flexion	**-2.01 (10.84)^c, d^**	71.38 (55.94)	**15.66 (24.92)^c^**	72.64 (12.24)	**16.91 (16.79)^d^**	73.45 (11.97)
Min. Palmar Flexion	-19.10 (5.41)	59.41 (53.51)	-16.24 (15.04)	36.54 (14.83)	-19.43 (13.27)	22.14 (31.51)
Range	**14.98 (6.25)^a, b^**		**33.46 (27.60)^a^**		**37.58 (27.80)^b^**	
						
***Trunk***						
Max. Flexion	-0.79 (7.03)	29.54 (28.50)	3.62 (18.66)	41.80 (58.28)	3.20 (15.66)	55.41 (33.10)
Min. Flexion	-7.43 (11.84)	58.36 (22.15)	-7.58 (14.10)	51.07 (16.99)	-8.82 (9.87)	50.61 (13.38)
Range	6.38 (5.54)		7.86 (10.59)		11.37 (6.77)	
						
Max. Lat. Incl.	5.01 (4.77)	57.34 (37.10)	5.21 (6.76)	65.45 (41.17)	10.28 (9.76)	48.34 (38.71)
Min. Lat. Incl.	2.82 (4.18)	63.99 (36.20)	2.50 (4.79)	35.42 (63.64)	6.17 (10.98)	24.26 (54.68)
Range	2.25 (1.93)		3.17 (5.26)		4.21 (3.52)	

a, b (p < 0.05, with Bonferroni correction)

c, d (p < 0.01, with Bonferroni correction)

Abbreviations: IR - Interquartile Range

% mov time-% of movement time

**Figure 4:**
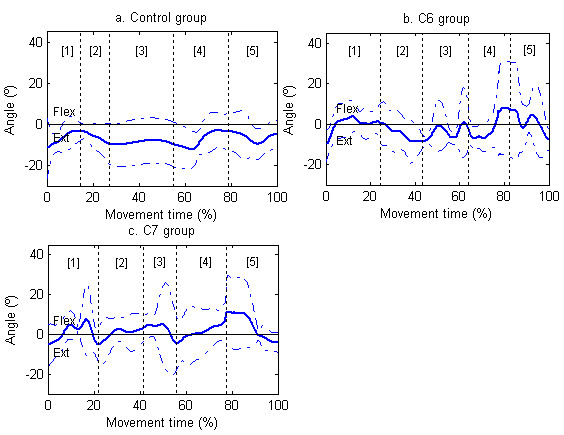
**Wrist dorsal-palmar flexion**. Joint angles for wrist (dorsal flexion-downward, palmar flexion-upward). Figures 4a, 4b and 4c show the mean (continue thick line) and standard deviation (dashed line) of the CG, subjects with C6 tetraplegia and subjects with C7 tetraplegia, respectively. The vertical lines delimit the duration of the phases for each group [1] reaching, [2] forward transport, [3] drinking, [4] back transport, and [5] return to beginning

The wrist was the joint in which the most relevant differences were found. The wrist palmar flexion angle was greater in the two tetraplegia groups than in CG (p < 0.01), which made the ROM of wrist dorsal-palmar flexion smaller in CG than in the subjects with C6 (p < 0.01) and C7 tetraplegia (p < 0.05). In CG, wrist palmar flexion values were not even found in the cycle (Table 3). The maximum wrist palmar flexion in the three groups was found in the back transport phase (at the moment corresponding to 72.64% of the cycle of subjects with C6 tetraplegia, 73.45% of the cycle of subjects with C7 tetraplegia and 71.38% of the cycle of CG) (Figure 4). On the other hand, minimum wrist palmar flexion in subjects with C6 or C7 tetraplegia was in the forward transport phase (at 36.54% and 22.14% of the cycle, respectively), and in CG it was in the back transport phase (at 59.41% of the cycle) (Table 3).

### Interjoint coordination between the shoulder and elbow

**Figure 5:**
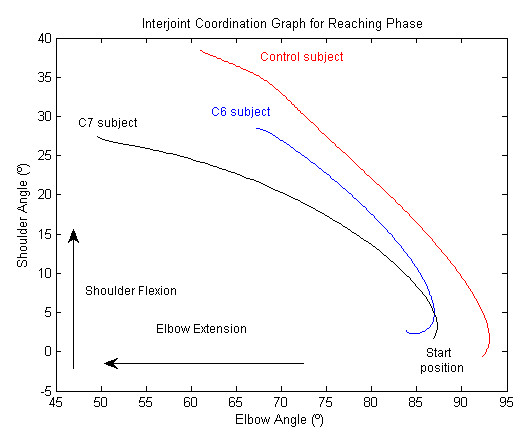
**Shoulder-elbow joint coordination in the reaching phase for one randomly selected subject in the control group (red), C6 group (blue) and C7 group(black)**.

In the three study groups, there was a strong coordination between the shoulder and elbow joint angles. The Pearson correlation index ranged from -0.95 (IR 0.08) to -0.91 (IR 0.11) (Table 2). The negative value of the correlation index meant that as shoulder flexion increased, elbow extension also increased. The trajectory of these correlations was continuous, forming an almost linear relation between the shoulder and elbow joint angles (Figure 5).

### Test-retest consistency

The statistical results of ten variables are shown in Table 4. Mean retest values were within for the 95% confidence interval of the first test. Based on this data, we concluded that there were no differences between the test and retest with a probability of 95%. However, particularly for measures as maximum shoulder flexion, maximum external rotation, maximum elbow flexion, maximum pronation, even maximum wrist palmar flexion, wide confidence intervals were obtained.

**Table 4:** Test-retest consistency for ten kinematic variables in 4 control subjects.

	Mean difference (95% CI)	CI (95%) of mean difference	T value	p value
Max. Shoulder Flexion (°)	-2.60	-19.37,13.95	-0.50	0.65
Max. Shoulder Abduction (°)	-1.02	-7.54,5.50	-0.49	0.65
Max. External Rotation (°)	-4.34	-12.87,18.20	-1.62	0.20
Max. Elbow Flexion (°)	-2.48	-19.37,14.40	-0.46	0.67
Max. Pronation (°)	2.53	-12.33,17.40	0.54	0.62
Max. Wrist Palmar Flexion (°)	-0.90	-10.30,8.49	-0.30	0.77
Max. Trunk Flexion (°)	-1.67	-5.41,2.06	-1.42	0.25
Max. Trunk Lateral Inclination (°)	-0.49	-3.11,2.12	-0.60	0.59
Movement time (s)	-0.21	-0.65,0.22	-1.54	0.22
Peak velocity in reaching (m/s)	-0.03	-0.09,0.03	-1.70	0.19

Abbreviations: CI - Confidence Interval

Value of the T statistic (Student t test).

## Discussion

The goal of this study was to analyze the three-dimensional kinematic differences between two groups of people with tetraplegia and a control group during the ADL of drinking from a glass. The most relevant findings of this study suggest that subjects with C6 tetraplegia perform the drinking task at a slower velocity and with more prolonged phases. The greatest differences between the two tetraplegia groups and controls were in the wrist. A few studies have been made of the kinematic properties of the arms of patients with tetraplegia, but none of them has analyzed an ADL [24,30,31]. The slower velocity of subjects with C6 tetraplegia when executing the drinking task coincides with the findings of previous studies that report that patients with C6 tetraplegia were slower than control subjects in performing pointing movements on the horizontal plane [30,32]. On the other hand, Wierzbicka et al. observed that the fast elbow flexion movement, due to the lack of an antagonist, had an important effect on completion time of fast goal-directed movements [31,33]. Finally, Laffont et al. concluded that in spite of some quantitative differences, the kinematics of the hand during reaching and pointing in quadriplegic patients are surprisingly similar to those of control subjects [24].

However, more functional movements should be studied. Previous studies of upper limb kinematics have been made of control subjects performing ADLs such as feeding, grooming and drinking [7,19,34,35]. These movements are complex tasks in terms of kinematics because they consist of several discrete movements. Studies have analyzed upper limb kinematics in certain specific groups, such as a normal pediatric population [20] or groups of patients with conditions like cerebral palsy or distal radius fracture performing certain functional activities [22,36].

Much of the methodology developed in the present study followed the recommendations of a previous one of healthy subjects in which five sequential phases of drinking task were identified: reaching, forward transport, drinking, back transport and returning [7]. However, the current experience has resolved previous limitations and provides a full and detailed three-dimensional kinematic analysis of the drinking task in control subjects and two groups of patients with tetraplegia, analyzing the shoulder, elbow and wrist at all possible joint angles except for lateral wrist inclination.

Using the upper limb model developed, we were able to estimate the location of the center of the joints involved, which made it possible to measure all the joint angles described. Likewise, the use of markers mounted on rigid pieces to position some of the markers helped to reduce tissue artifacts. These artifacts appear with limb displacement when markers are placed on the skin surface.

It has been reported that trunk movement can act as both a stabilizer and an integral component in positioning the hand close to the target [37]. It has been shown that hemiparetic subjects reaching within arm's length use a compensatory strategy that involves trunk displacement [38,39]. In the present study, the glass was placed within arm's length and the subject could reach it without separating the trunk from the back of the wheelchair. Our findings confirmed those of earlier experience carried out in control subjects, in which trunk displacement was not relevant in the groups analyzed [36].

### 1. Movement times

The total duration of the drinking task was somewhat shorter in our CG than in an earlier report, probably because in the present study the palm of the hand was closer to the drinking glass whereas in the earlier report the wrist line was closer to the edge of the table [7]. However, both two studies had the same conclusion: back transport is the most prolonged phase in controls [7]. The duration of the drinking activity was longer in subjects with C6 tetraplegia compared to controls and the duration of the reaching phase was longer in subjects with C6 and C7 tetraplegia. As mentioned, the reaching phase includes grasping. In order to grasp, both groups of patients with tetraplegia developed a compensatory strategy called "tenodesis," in which these patients extend the wrist to close the fingers passively. This pattern suggests that in subjects with tetraplegia reaching and grasping are executed sequentially compared to controls, who prepare for grasping during the reaching phase [40].

### 2. Peak velocity

As the duration of the drinking task was shorter, the velocity of each phase of the cycle in the controls was somewhat faster than in a previous report [7]. The absence of triceps brachialis muscle activity in subjects with C6 tetraplegia slows the velocity of the forward transport and back transport phases, in which this muscle controls the eccentric or concentric displacement of the elbow in flexion-extension. As in an earlier study, the peak velocity of the reaching phase was similar in patients with tetraplegia and controls [24]. Another factor that could condition the velocity of movements is performing the movement with a load. The weakness of the upper limbs becomes more evident when raising an object with a certain weight. In the absence of any additional load, peak velocity in the reaching phase is reached earlier in groups of patients with tetraplegia. However, in the forward transport phase in which the glass of water is raised to the mouth, peak velocity is notably faster in controls. It is difficult to compare the velocities attained in other pathologies because they have not been studied using the phases defined in our study [23].

### 3. Joint angles

As in healthy subjects, but in contrast with subjects who have experienced stroke and have a hemiparetic arm, there was a strong coordination between shoulder and elbow joint excursion in the reaching phase, indicating good interjoint coordination in C6 and C7 tetraplegia [10,12]. The wrist was the joint with the most relevant differences between the three groups. Wrist palmar flexion angles were greater in both groups of subjects with tetraplegia and the maximum wrist palmar flexion in both cases was observed in the back transport phase, probably because no eccentric resistance is offered by wrist extensor muscles as the glass is lowered from the mouth to the table; passive wrist palmar flexion occurred in both tetraplegia groups. The minimum wrist palmar flexion angle was found in subjects with C6 or C7 tetraplegia in the forward transport phase. This is probably because at this time the subject required maximum wrist dorsal flexion to grasp a glass that has some weight, which optimized the tenodesis effect and the ability to pick up an object. The elbow extension was greater in both tetraplegia groups and occurred in the back transport phase, perhaps also because elbow extension favored the tenodesis effect in the wrist.

### 4. Test-retest consistency

Mean retest values were within for the 95% confidence interval of the first test. Based on this data, we concluded that there were not differences between the test and retest with a probability of 95%. However, for measurements as maximum shoulder flexion, maximum external rotation, maximum elbow flexion, maximum pronation, even maximum wrist palmar flexion, wide confidence intervals were obtained. It could be probably due to the natural large variation between the subjects in those measurements. It is necessary to take into account that people can perform a goal-oriented task with many different combinations of individual joint movements.

However, although the results obtained were sound, for further research in this field, it would be good to include another scanning unit to offer a view from above with the aim of providing the best visibility of the markers in the three space planes.

Robotic tools provide opportunities to study functional adaptation after central nervous system injuries and can provide objective measurements of the time-course of changes in motor control of the affected limbs. Robot-assisted therapy permits semi-autonomous practice of therapeutic tasks [41]. In the last years, wearable technology has made an impact in the clinical setting and recent studies have been focused on integrating this technology with orthotic and prosthetic devices [42]. So, wearable robots development can be one of the most innovative therapeutic options for people with cervical SCI, to improve upper limb functionality and so, facilitate the independence and quality of life during the performance of ADL. In order to design these devices, it is necessary to identify the movement patterns performed by these patients during functional activities, such as the drinking task. Then, these movement patterns can be implemented into robotic devices to imitate or improve these movements. In this research field, the present study is of particular clinical relevance.

## Conclusions

Kinematic analysis has shown great potential for use as an outcome in clinical research to understand how functional activities, such as drinking, are performed by patients with upper limb impairment. The most relevant differences were in the wrist, where the palmar flexion values were greater in patients with C6 and C7 tetraplegia than in controls during the back transport phase, whereas the highest wrist dorsal flexion value was in the forward transport phase in subjects with C6 or C7 tetraplegia, in which complete activation of the tenodesis effect is needed for grasping. This information can be useful in designing wearable robots to compensate the performance of AVD, such as drinking, in people with cervical SCI.

## Competing interests

None of the authors of this paper has any conflict of interest in relation to any sources of any kind pertinent to this study.

## Authors' contributions

ARG contributed to the concept and design, planning of study, software development, analysis and interpretation of the data, drafting and completion of the manuscript. AGA contributed to design, analysis of the data and completion of the manuscript. BPM contributed to the concept, software development, design and acquisition of the data. MSM contributed to the analysis and acquisition of the data. AAE contributed to the analysis and acquisition of the data. EPR contributed to the software development. All authors read and approved the manuscript to be published.
